# Attrition from HIV care among youth initiating ART in youth‐only clinics compared with general primary healthcare clinics in Khayelitsha, South Africa: a matched propensity score analysis

**DOI:** 10.1002/jia2.25854

**Published:** 2022-01-25

**Authors:** Tali Cassidy, Morna Cornell, Pumeza Runeyi, Thembie Dutyulwa, Charllen Kilani, Laura Trivino Duran, Nompumelelo Zokufa, Virginia de Azevedo, Andrew Boulle, C. Robert Horsburgh, Matthew P. Fox

**Affiliations:** ^1^ Médecins Sans Frontières Cape Town South Africa; ^2^ Division of Public Health Medicine School of Public Health and Family Medicine University of Cape Town Cape Town South Africa; ^3^ Department of Epidemiology Boston University School of Public Health Boston Massachusetts USA; ^4^ Centre for Infectious Disease Epidemiology & Research School of Public Health and Family Medicine University of Cape Town Cape Town South Africa; ^5^ City Health Department Cape Town South Africa; ^6^ Western Cape Provincial Department of Health Western Cape South Africa; ^7^ Section of Infectious Diseases Department of Medicine Boston University School of Medicine Boston Massachusetts USA; ^8^ Department of Global Health Boston University School of Public Health Boston Massachusetts USA; ^9^ Health Economics and Epidemiology Research Office Faculty of Health Sciences Department of Internal Medicine School of Clinical Medicine University of the Witwatersrand Johannesburg South Africa

**Keywords:** HIV, antiretroviral therapy, retention in care, youth, differentiated service delivery

## Abstract

**Introduction:**

Youth living with HIV (YLWH) are less likely to initiate antiretroviral therapy (ART) and remain in care than older adults. It is important to identify effective strategies to address the needs of this growing population and prevent attrition from HIV care. Since 2008, two clinics have offered youth‐targeted services exclusively to youth aged 12–25 in Khayelitsha, a high HIV‐prevalence, low‐income area in South Africa. We compared ART attrition among youth in these two clinics to youth in regular clinics in the same area.

**Methods:**

We conducted a propensity score matched cohort study of individuals aged 12–25 years initiating ART at eight primary care clinics in Khayelitsha between 1 January 2008 and 1 April 2018. We compared attrition, defined as death or loss to follow‐up, between those attending two youth clinics and those attending general primary healthcare clinics, using Cox proportional hazards regression. Follow‐up time began at ART initiation and ended at attrition, clinic transfer or dataset closure. We conducted sub‐analyses of patients attending adherence clubs.

**Results:**

The distribution of age, sex and CD4 count at ART initiation was similar across Youth Clinic A (*N* = 1383), Youth Clinic B (*N* = 1299) and general clinics (*N* = 3056). Youth at youth clinics were more likely than those at general clinics to have initiated ART before August 2011 (Youth Clinic A: 16%, Youth Clinic B: 23% and general clinics: 11%). Youth clinics were protective against attrition: HR 0.81 (95% CI: 0.71–0.92) for Youth Clinic A and 0.85 (0.74–0.98) for Youth Clinic B, compared to general clinics. Youth Clinic A club patients had lower attrition after joining an adherence club than general clinic patients in adherence clubs (crude HR: 0.56, 95% CI: 0.32–0.96; adjusted HR: 0.48, 95% CI: 0.28–0.85), while Youth Clinic B showed no effect (crude HR: 0.83, 95% CI: 0.48–1.45; adjusted HR: 1.07, 95% CI: 0.60–1.90).

**Conclusions:**

YLWH were more likely to be retained in ART care in two different youth‐targeted clinics compared to general clinics in the same area. Our findings suggest that multiple approaches to making clinics more youth‐friendly can contribute to improving retention in this important group.

## 1 INTRODUCTION

In 2017, HIV prevalence among South African youth aged 15–25 was 7.1% [1], with an annual incidence of 1% [2]. Among youth living with HIV (YLWH) aged 15–24, just 40% were on antiretroviral therapy (ART) in 2017, compared to 63% of HIV‐positive adults aged 25–49 [3]. A review of adolescent HIV care in South Africa identified six studies measuring retention in care, all of which showed poorer retention of youth compared to adults (ORs 1.55–2.25) [4]. Poorer youth retention has been observed throughout the region: a large cohort analysis of ART patients in sub‐Saharan Africa found youth (15–25) to have a higher risk of attrition than older adults [5]. ART care improves outcomes of people living with HIV and reduces transmission [6, 7, 8, 9]. As youth on ART are a growing population[10], it is important to identify effective strategies to address youth‐specific needs and prevent attrition from care.

Strategies to make ART services more “youth‐friendly” and facilitate the transition from child to adult care have included counselling and support services, peer support, dedicated youth times and staff sensitization [11, 12, 13, 14]. Evaluations of such interventions have yielded mixed results, and as these are often implemented together in a single service, it is difficult to disentangle the relative importance of each element of youth‐friendly services. A 2019 review identified two interventions associated with reduced youth attrition [14]. A dedicated adolescent clinic (*n* = 88) showed improved retention in care compared to standard clinic care in a South African setting [15]. In a Malawian hospital, attending a teen club provided adolescents (10–19 years old) with dedicated clinic time, services and peer support, and was associated with a large decrease in attrition (OR: 0.27) [16]. A Zimbabwean study randomized an intensive treatment support program, including treatment supporters, support groups, text messages, calls, home visits and clinic‐based counselling. Adolescents in intervention clinics had improved virological outcomes (prevalence ratio: 0.58) and attendance (prevalence ratio: 0.80), suggesting that peer support and enhanced counselling play an important role in youth retention and ART adherence [17]. However, a Kenyan intervention, which included support groups, peer education and dedicated clinic time, failed to show an improvement in youth ART outcomes [18].

Two youth clinics (referred to here as Youth Clinic A and Youth Clinic B) in Khayelitsha, South Africa, specifically aim to attract and retain youth in care. YLWH (aged 12–25) are a growing population who will potentially be on ART for many years. Reducing attrition in this group is important to reduce mortality, transmission and drug resistance. Through slightly different models, both Khayelitsha youth clinics attempt to address some of the psychosocial and provider‐related causes of youth attrition.

## 2 METHODS

### 2.1 Study design

Using routine clinical records, we conducted a propensity score matched longitudinal analysis of individuals aged 12–25 years at primary care clinics that provide HIV care services to youth in Khayelitsha, looking at the outcome of attrition from care. We also conducted a sub‐analysis of adherence club patients comparing attrition in Youth Clinic A and B to the reference category of general clinics, from the time of club enrolment.

### 2.2 Population and setting

The City of Cape Town's Health department offers HIV care at two youth‐only clinics and six general primary healthcare clinics in Khayelitsha, a low‐income, high HIV‐prevalence, peri‐urban area in South Africa, home to approximately 500,000 people [19, 20].

We included YLWH aged 12–25 years who first initiated ART between 1 January 2008 and 1 April 2018 at any of these eight clinics. Data on time of HIV infection were not available, but it is assumed that perinatal infections constitute a small proportion of those initiating ART aged 12–25 in this context. Patients who had tuberculosis at ART initiation were excluded because these patients were often referred from youth clinics to general clinics.

### 2.3 Clinic services

Both youth clinics provide primary healthcare and HIV services exclusively to youth between 12 and 25 years old. Since 2008, both youth clinics have offered ART to eligible YLWH (Table 1).

**Table 1:** Summary of characteristics and services of Youth Clinic A, Youth Clinic B and general clinics

	Youth Clinic A	Youth Clinic B	General clinics
Target population	Exclusively youth between 12 and 25 years old		All age groups
HIV services	HIV testing, counselling, ART initiation and management		
Other services	Contraception, TB and STI screening and basic curative services		Contraception, TB and STI screening and basic curative services TB treatment initiation and management
Counselling	Counselling before and after HIV testing, and adherence counselling is provided by lay adherence counsellors, a key cadre supporting the retention of ART patients [21, 22, 23] who are managed by non‐profit organizations at all clinics in Khayelitsha		
Health authority	City of Cape Town Department of Health		
Adherence clubs	Peer support from other youth, differentiating between youth of school‐going age and those over the age of 18 Can be joined immediately after ART initiation Integrated family planning services	Peer support from other youth Eligible 6–12 months after initiation	Peer support from other patients (not differentiated by age) Eligible 6–12 months after initiation
Transition	Transfer to general clinic when turning 26, usually main clinic on same premises (see “structure” below)	Transfer to general clinic when turning 26, usually main clinic on nearby premises (see “structure” below)	Not needed
Visits	Visits are typically spaced 1 month apart when patients initiate and 2 months apart for stable patients and patients in adherence clubs. Before the holiday period (December–January), patients may receive 4 months of medication.		
Pharmacy	For patients not in adherence clubs, ART is collected from an on site pharmacy after their clinical visit.		
Records	Captured into PREHMIS and electronic medical records system from patient folders after patients attend a visit. Folders are stored on site and captured by data clerks at the clinics.		
Structure	Standalone building on premises of larger clinic	Standalone building on premises near larger clinic	Standalone buildings on clinc premises
Geographic location (all within Khayelitsha)	Khayelitsha Located in Site C, a dense, largely informal area	Khayelitsha Located in Site B, a dense, largely informal area	Khayelitsha Variety of areas across Khayelitsha
Médecins Sans Frontières involvement 2008–2010	Supported	Supported	Not specifically supporting youth services
Médecins Sans Frontières involvement after 2010	Managed counsellors	Handed over	Not specifically supporting youth services

In Khayelitsha clinics, many stable ART patients receive their medication in adherence clubs, a differentiated service delivery model led by a lay facilitator. To be eligible to join and remain in an adherence club, patients are required to be stable on ART and have a suppressed HIV viral load [24, 25]. This model has proven to be acceptable, scalable and effective, while also providing peer support [26, 27, 28, 29, 30, 31, 32, 33, 34]. In general clinics, youth are typically in clubs with people of all ages. In youth clinics, peer support from other youth might enhance the benefit of clubs.

Youth Clinic A further divides clubs by age, as youth of different ages may have different challenges. When appropriate, youth transition from younger to older adherence clubs, reducing the difficulty of transitioning to adult care [11, 12, 13]. Youth Clinic A adherence clubs include family planning services. Integration of other services and social support have been identified as potential ways of addressing youth‐specific needs [35, 36]. At Youth Clinic A, adherence clubs can be joined immediately after ART initiation, unlike typical adherence clubs, which may only begin after 6–12 months. Stable ART patients receive medication refills in the group, while newly initiated youth receive refills from a nurse during a clinical consultation on the same day [37, 38].

The youth clinics were initially supported by Médecins Sans Frontières (MSF). In 2010, the City of Cape Town's health department took over the ART services at both clinics, although MSF continued support Youth Clinic A, primarily through lay counsellors who were managed by MSF and trained to be sensitive to the needs of youth [38].

### 2.4 Data source

Data for this study were routinely captured into an electronic medical records system by clinic data clerks. Data were captured from standardized clinical stationery in patient folders after patients attended each of their ART visits, throughout the follow‐up period.

### 2.5 Measures

We investigated the effect of receiving HIV treatment from one of two youth clinics compared with receiving care at a general clinic on attrition, defined as loss to follow‐up or death. Deaths were not actively ascertained, so we assumed, as reported in other contexts [39, 40, 41], that deaths were misclassified as loss to follow‐up in clinic data. Loss to follow‐up was automatically recorded in the clinics’ electronic data system when a patient missed a scheduled visit by more than 3 months. If a patient had a subsequent visit any time during the follow‐up period, they would no longer reflect as lost to follow‐up. Transfers were ascertained either if recorded at the clinic of initiation or if they were recorded as re‐entering care under the same patient identifier at another clinic included in this dataset. Baseline CD4 count was the closest available result to the date of ART initiation, between 180 days before and 30 days after initiation.

### 2.6 Follow‐up time

The dataset was closed on 30 September 2018. For time‐to‐event analyses, follow‐up time for each person started at date of ART initiation and ended on the last attended visit before 1 April 2018, regardless of their outcome. A patient's outcome was their status as of dataset closure: if a patient was lost to follow‐up but returned before dataset closure, they would be considered retained in care. Patients who transferred out were censored on their last visit date at their original clinic, even if the transfer was to another clinic in this dataset. Patients in care were censored on their 26th birthday, as this is when they would have been required to transfer out of the youth clinic. If a patient's last attended visit was the same as their ART initiation date, 1 day of follow‐up was added to allow for inclusion in time‐to‐event analyses (see Table S8 for a sensitivity analysis of this approach) [42].

### 2.7 Analysis

#### 2.7.1 Multiple imputation

Missing values of baseline disease stage, CD4 count and regimen were assumed to be missing at random and imputed using gender, age, ART start date and era, attrition and clinic type as predictor variables (Table S1 summarizes missing data). Chained multiple imputation [43, 44, 45] was used to create 20 imputed datasets using the *ice* procedure in Stata [46, 47].

#### 2.7.2 Propensity score matched analysis

Propensity score matching was used to create a group of general clinic patients comparable with youth clinic patients [48, 49]. Separate analyses were conducted for each youth clinic: Youth Clinic B was excluded from the Youth Clinic A analysis, and vice versa. For each analysis, propensity scores were generated using logistic regression, with clinic type (youth vs. general clinic) as the dependent variable. Independent variables were ART initiation date and WHO Disease Stage as these were associated with attrition and clinic type. Propensity scores were generated for each of the imputed datasets and an average propensity score for each observation was calculated across datasets [50]. Each youth clinic patient was matched 1:1 to a general clinic patient based on their propensity score, using nearest neighbour matching without replacement.

We performed a Cox proportional hazards regression on both matched datasets to estimate the hazard ratio (HR) of attrition associated with attending the respective youth clinic compared to general clinics, using robust standard errors specifying clustering by matched pair [51, 52, 53, 54]. The multiple imputed datasets were combined using Rubin's methods [55]. To adjust for any residual confounding after propensity score matching, covariates were individually added to the model, but none changed the HR by more than 10% (Table S7). E‐values were calculated using the *EVALUE* module in Stata 14 [56].

Proportional hazards assumption was assessed with chi‐squared goodness of fit tests (Tables S4 and S5). All analyses were conducted in Stata 14 [44].

#### 2.7.3 Adherence club analysis

We conducted a sub‐analysis of patients who attended an adherence club before the age of 26. We compared overall attrition from the time of first adherence club visit in a Cox proportional hazards model, by clinic type (Youth Clinic A and B, compared to general clinics).

#### 2.7.4 Sensitivity analyses

We performed several sensitivity analyses. First, ART guideline era, as a categorical variable, was included in the propensity score models instead of ART initiation date. Shifts in ART eligibility and other secular trends are adjusted for in the main analysis by including ART initiation date in the propensity score model, but it is possible that the guideline era of ART initiation is a better measure of this confounder. Second, we used the full unmatched dataset to estimate the effect of clinic type, defined as a multi‐level exposure (Youth Clinic A and B, compared to general clinics). We estimated the crude HRs, HRs adjusted for ART start date and HRs adjusted for ART start date and WHO Stage. Finally, a secondary outcome definition was used where a patient was considered lost to follow‐up the first time there was a 9‐month gap in care, even if they return to care at a later date, with the last visit before the gap being the outcome date.

### 2.8 Ethics

Ethics approval was granted by the University of Cape Town's Human Research Ethics Committee (HREC395/2005), who waived consent as the analyses used de‐identified data collected as part of routine patient care.

## 3 RESULTS

**Figure 1:**
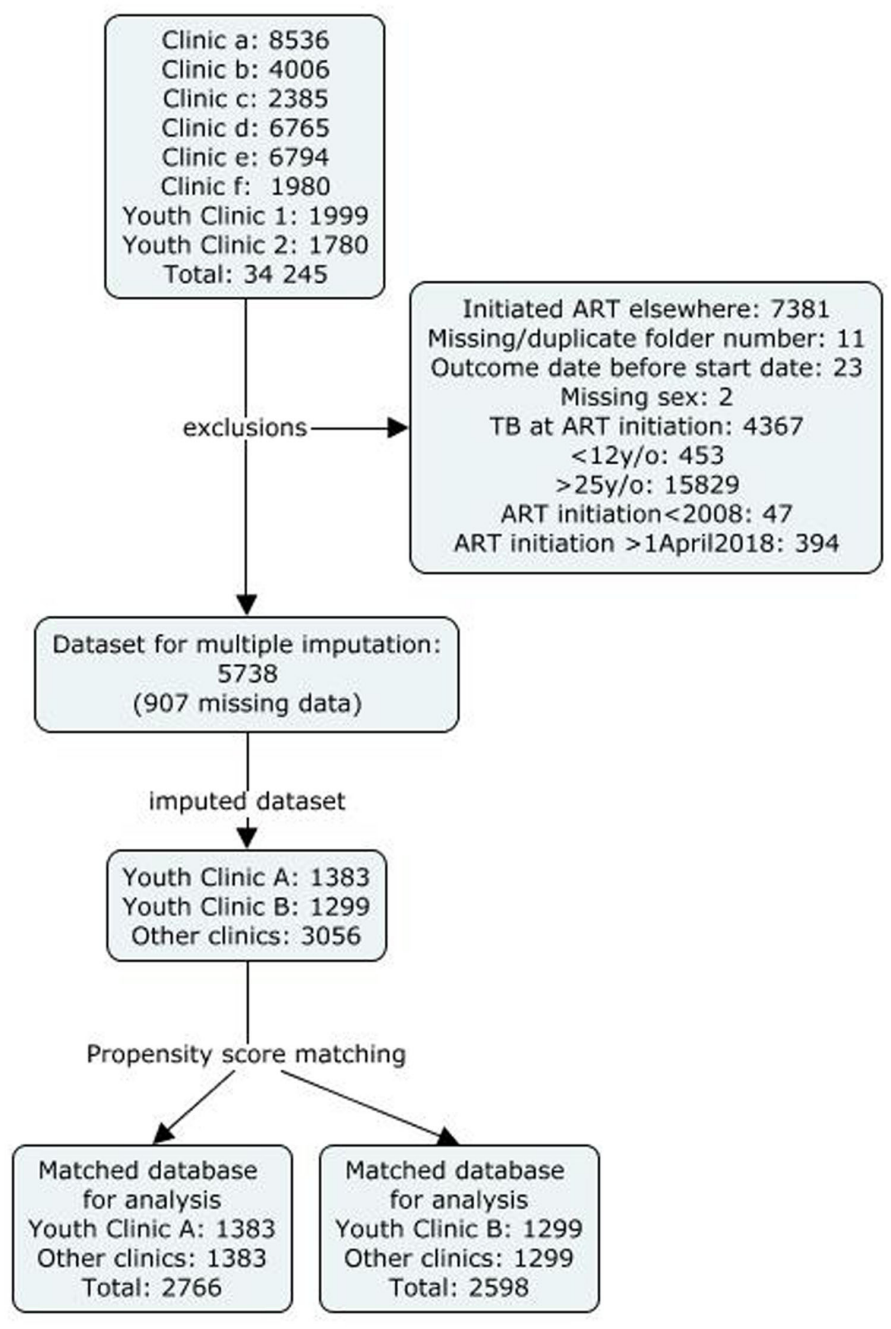
Flow chart for inclusion in analysis.

We included 5738 YLWH in this analysis: 1383 from Youth Clinic A, 1299 from Youth Clinic B and 3056 eligible comparisons from other clinics (Figure 1), making the analysis well‐powered to detect a protective effect below HR = 0.82 (Figure S3).

Youth at youth clinics were more likely than those at general clinics to initiate ART before August 2011 (Youth Clinic A: 16%, Youth Clinic B: 23% and general clinics: 11%). The median age of ART initiation was similar across Youth Clinic A (22.2; IQR: 20.4–23.5), Youth Clinic B (21.8; IQR: 20–23.3) and general clinics (22.4; IQR: 20.4–23.8), as was the proportion of males (Youth Clinic A and general clinics: 11%, Youth Clinic B: 9%). General clinics had a slightly higher proportion of patients initiating ART at WHO Disease Stages 2–4 (29%, Youth Clinic A: 20%,Youth Clinic B: 25%). CD4 count at ART initiation was similar across clinics (Table 2).

**Table 2:** Distribution of covariates by clinic group, after imputation (before and after propensity score matching)

	General clinics (before matching)	Youth Clinic A	Matched controls	Youth Clinic B	Matched controls
*N*	3056	1383	1383	1299	1299
Era of ART initiation					
Eligibility CD4<200 (before August 2011)	11%	16%	14%	23%	18%
Eligibility CD4<350 (August 2011–31 December 2014)	31%	31%	33%	31%	37%
Eligibility <500 (1 January 2015–31 August 2016)	29%	25%	24%	24%	24%
All eligible (1 September 2016)	28%	28%	28%	22%	22%
Age					
12–17 years	9%	8%	9%	9%	9%
18–25 years	91%	92%	91%	91%	91%
Median age (years) (IQR)	22.4 (20.4–23.8)	22.2 (20.4–23.5)	22.4 (20.4–23.8)	21.8 (20–23.3)	22.5 (20.4–23.8)
Sex					
Male	11%	11%	11%	9%	11%
Baseline WHO Stage					
Stage 1	71%	80%	82%	75%	77%
Stage 2	16%	14%	11%	11%	11%
Stage 3	10%	5%	5%	11%	10%
Stage 4	3%	1%	1%	3%	2%
Stages 2–4	29%	20%	18%	25%	23%
Baseline CD4 count (cells/mm^3^)					
<100	9%	6%	8%	9%	10%
100–199	15%	16%	16%	15%	16%
200–349	33%	40%	34%	36%	36%
350–500	24%	21%	23%	23%	21%
>500	19%	17%	20%	17%	17%
Median CD4 count (IQR)	318 (205–460)	303 (211–435)	315 (204–459)	310 (203–438)	304 (194–438.1)
ART regimen at initiation					
EFV‐free regimens	6%	9%	8%	11%	9%
TFV‐free regimens	9%	10%	10%	11%	12%
Adherence clubs					
Ever in club at clinic (before age 26)	19%	20%	19%	13%	18%
Median months in club (before age 26)	16.7 (6.3–25.8)	11.7 (4.6–21.1)		10.1 (1.8–20.5)	
Median months on ART before club	23.7 (12.7–45.8)	10.6 (3.6–20.1)		21.4 (14–36.8)	
% 6‐month attrition	21%	15%	20%	17%	20%

Abbreviations: EFV, Efavirenz; TFV, Tenofovir.

Before propensity score matching, overall 12‐month attrition was 25%. General clinics had somewhat higher attrition (27%) compared to Youth Clinic A (22%) and Youth Clinic B (24%). Those initiating ART after CD4 eligibility criteria were removed (September 2016) and had a 35% chance of 12‐month attrition, compared to 18% in those initiating ART before August 2011. This difference was reflected in all clinics, and greatest for general clinics (38% after September 2016 vs. 28% at Youth Clinic A and 35% at Youth Clinic B). Initiating treatment before the age of 18 was associated with slightly lower 12‐month attrition (22% vs. 26%) than initiating ART at older ages. Attrition at 12 months was lower among those initiating at WHO Disease Stage 1 (26%) compared to Stages 2–4 (19%); and also lower for those with CD4 counts of 100–200 cells/mm^3^ (19%) compared to higher CD4 counts and those <100 cells/mm^3^ (26%). Higher 12‐month attrition was observed in those initiating on regimens without Tenofovir (TFV) or Efavirenz (EFV) compared to those on both non‐TFV regimens and non‐EFV regimens (Table 3, Table S3 shows the breakdown by clinic type).

**Table 3:** Attrition from care at 6 and 12 months by covariates (without imputation or propensity score matching)

	Attrition by 6 months	Attrition by 12 months
	*N* = 5157^a^	*N* = 4540^b^
Total	19%	25%
Exposure group		
General clinics	21%	27%
Youth Clinic A	15%	22%
Youth Clinic B	17%	24%
Sex		
Male	18%	24%
Female	19%	25%
Era of ART initiation (by CD4 count eligibility criteria)		
<200 (before August 2011)	11%	18%
<350 (August 2011–31 December 2014)	16%	22%
<500 (1 January 2015–31 August 2016)	19%	27%
All eligible (after 1 September 2016)	28%	35%
Age		
12–17 years	16%	22%
18–25 years	19%	26%
WHO Stage at initiation		
Stage 1	19%	26%
Stage 2	14%	18%
Stage 3	14%	19%
Stage 4	23%	28%
Stages 2–4	15%	19%
Stage missing	29%	36%
Baseline CD4 count (cells/mm^3^)		
<100	17%	26%
100–199	15%	19%
200–349	17%	23%
350–500	21%	29%
>500	23%	30%
CD4 count missing	19%	28%
ART regimen at initiation		
EFV‐free regimens	11%	15%
TFV‐free regimens	12%	17%
TFV‐EFV regimens	19%	26%
Missing regimen information	29%	38%
Ever in adherence club at clinic	0%	2%
Never in adherence club at clinic	23%	31%

^a^ 6‐month retention is only presented for those who initiate ART more than 9 months before dataset closure.

^b^ 12‐month retention is only presented for those who initiate ART more than 15 months before dataset closure.

### 3.1 Propensity score matched cohort analysis

Propensity score matching reduced the association between clinic type and ART initiation era, and clinic type and WHO Stage (Table 2, see Figure S1 for propensity score distributions and Figure S2 for standardized differences). After matching, 6‐month attrition was substantively higher among general clinic patients compared to youth clinic patients (Table 3).

The matched propensity score cohort for Youth Clinic A included 833 events over 4367 person‐years and the Youth Clinic B analysis included 804 events over 4341 person‐years (Tables S4 and S5). In the matched cohorts, 7% of patients had only their first visit, and 4% were immediately lost to follow‐up. This was similar across clinics. More patients in general clinics had a 9‐month gap in care, and more general clinic patients met this outcome definition and subsequently returned to care (10% overall, Table S6).

**Figure 2:**
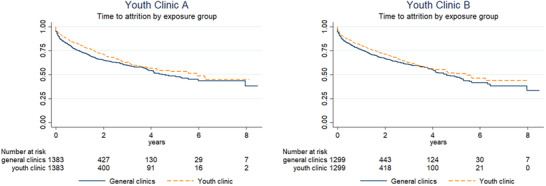
Kaplan–Meier survival estimates for matched cohorts, by clinic type.

Kaplan–Meier curves for the matched cohorts (Figure 2) show lower risk of attrition for both youth clinics compared to general clinics.

Table 4 shows HRs from matched propensity score models, where ART start date and WHO Disease Stage are included in the propensity score model. Compared to propensity score matched general clinic patients, there is a protective effect of youth clinics against attrition: HR 0.81 (95% CI: 0.71–0.92) for Youth Clinic A and 0.85 (0.74–0.98) for Youth Clinic B.

**Table 4:** Cox regression models of attrition risk of youth clinic compared to general clinic patients

Method	Variables adjusted for/included in propensity score model	HR of risk of attrition in youth clinic versus general clinics (95% CI)	
		Youth Clinic A	Youth Clinic B
Full cohort analyses		*N* = 2766	*N* = 2598
Matched propensity score approach^a^	Start date and WHO Disease Stage	0.81 (0.71–0.92)	0.85 (0.74–0.98)
	Guideline era and WHO Disease Stage	0.74 (0.65–0.84)	0.90 (0.78–1.03)
No matching, combined Cox model, including Youth Clinic A and B^b^	Crude	0.78 (0.69–0.88)	0.80 (0.71–0.90)
	Adjusting for start date	0.80 (0.71–0.91)	0.86 (0.76–0.97)
	Adjusting for ART start date, WHO Stage	0.81 (0.72–0.92)	0.87 (0.77–0.98)
*Secondary definition of outcome* ^c^			
Matched propensity score approach^a^	Start date and WHO Disease Stage	0.68 (0.60–0.76)	0.72 (0.64–0.81)
	Guideline era and WHO Disease Stage	0.62 (0.55–0.70)	0.75 (0.66–0.85)
No matching, combined Cox model, including Youth Clinic A and B^a^	Crude	0.66 (0.59–0.73)	0.68 (0.61–0.75)
	Adjusting for start date	0.67 (0.60–0.75)	0.71 (0.64–0.79)
	Adjusting for ART start date, WHO Stage	0.68 (0.61–0.75)	0.72 (0.64–0.80)
Adherence club patients only (*N* = 912)			
No matching, combined Cox model, including Youth Clinic A and B^b^	Crude	0.56 (0.32–0.96)	0.83 (0.48–1.45)
	Adjusting for start date	0.49 (0.28–0.84)	0.98 (0.56–1.72)
	Adjusting for ART start date, WHO Stage	0.49 (0.28–0.85)	0.98 (0.56–1.72)
	Adjusting for ART start date, WHO Stage and age at club start	0.50 (0.29–0.88)	1.00 (0.57–1.77)
	Adjusting for ART start date, WHO Stage, age at club start and time on ART at club start	0.48 (0.28–0.85)	1.07 (0.60–1.90)
*Secondary definition of outcome* ^c^			
No matching, combined Cox model, including Youth Clinic A and B^b^	Crude	0.50 (0.30–0.85)	0.58 (0.32–1.06)
	Adjusting for start date	0.48 (0.28–0.83)	0.61 (0.33–1.12)
	Adjusting for ART start date, WHO Stage	0.49 (0.29–0.85)	0.60 (0.33–1.10)
	Adjusting for ART start date, WHO Stage and age at club start	0.49 (0.28–0.84)	0.60 (0.32–1.09)
	Adjusting for ART start date, WHO Stage, age at club start and time on ART at club start	0.48 (0.28–0.82)	0.63 (0.34–1.17)

^a^ Analyses conducted separately for Youth Clinic A and B.

^b^ Analysis of full dataset, including Youth Clinic A and B, with general clinics as reference group.

^c^ The first time there is a 9‐month gap in care patients are considered lost to follow‐up even if they return to care, with the date of last visit before the gap in care being the outcome date.

### 3.2 Sensitivity analyses

Results were similar when start date versus guideline era were included in the propensity score model. Results in the crude Cox models (no propensity score matching) were similar to the matched analyses, and adjustment for covariates resulted in little or no change to the crude HRs. The secondary definition of the outcome (a gap of nine months or more, regardless of subsequent visits) strengthened the protective effect of youth clinics against attrition, compared to general clinic patients, for all models (Table 4).

### 3.3 Adherence club analysis

At Youth Clinic A, 20% of patients were ever in an adherence club at the clinic, compared to 13% at Youth Clinic B and 19% at general clinics (Table 2 and Table S2).

Youth Clinic A club patients had lower attrition after joining an adherence club compared to general clinic patients (HR: 0.56, 95% CI: 0.32–0.96), and this protective effect strengthened after adjusting for ART start date, WHO Stage, age at club start and time on ART at club start (HR: 0.48, 95% CI: 0.28–0.85). Youth Clinic B club patients had similar attrition to general clinic club patients in all adjusted models using the primary outcome definition (Table 4).

### 3.4 E‐values

The E‐value was 0.6 for the primary Youth Clinic A analysis and 0.66 for Youth Clinic B. If there were an unmeasured confounder associated with both attrition and Youth Clinic A by a risk ratio of 0.6, then the true clinic‐attrition HR would be 1. (Tables S4 and S5 show all E‐values).

## 4 DISCUSSION

This study has demonstrated lower attrition among YLWH attending two youth clinics, serving only youth aged 12–25, compared to those attending general clinics in the same area. Youth Clinic A, located in a socio‐economically similar area to Youth Clinic B, showed a larger reduction in attrition than Youth Clinic B (compared to general clinics), suggesting that the added support in training youth‐friendly counsellors had an impact in this clinic.

The benefits of the youth clinic model, specifically observed at Youth Clinic A, may also be attributable to adherence clubs at youth clinics. Among adherence club patients, there was a large reduction in attrition in Youth Clinic A compared to general clinics. There were few adherence club patients at Youth Clinic A under 18 years old, so the observed effect is unlikely to be attributable to the club age segregation. Youth Clinic A patients were on ART for a shorter period at their first club visit, but adjusting for duration on ART strengthened the protective effect. Adherence club patients had similar attrition in Youth Clinic B and general clinics after adjusting for ART initiation era. These results should be interpreted with caution because of the small number (*n* = 133) ever in clubs at Youth Clinic B. However, the large improvement in outcomes at Youth Clinic A suggest that the model of care that integrates family planning within clubs and recruits patients into clubs earlier has added benefit above the social support provided by youth clubs.

Baseline characteristics were similar across clinics. Low proportions of males were seen at all clinics (9–11%), despite males accounting for 25% of South African youth aged 15–24 living with HIV in 2018 [1]. These low proportions are not explained by male youth initiating ART at two male‐only clinics in Khayelitsha in this time period, as this group was equivalent to just 2% of the 5738 youth included in Table 2. Among South African adults, ART coverage is lower among males (58%) than females (64%) [1], but this gap was particularly pronounced among youth in this analysis. Women typically have more opportunity to interact with the healthcare system [57, 58, 59, 60], and social and cultural barriers [61, 62, 63] might make men more resistant to seeking care. These factors might be particularly pertinent among young, otherwise healthy men. The similar sex distribution between youth and general clinics suggests that the youth clinics have not addressed these issues.

Overall 12‐month attrition was 25%, with higher attrition among patients initiating with lower baseline WHO Disease Stage, those on TFV‐EFV regimens and higher baseline CD4 counts. Our findings are consistent with other literature, suggesting that the benefit of ART may be less apparent to healthier people [64, 65]. This observation does not hold for those with CD4 counts below 100 cells/mm^3^, whose higher 12‐month attrition may be explained by higher death rates. The higher rates of attrition in those initiating more recently may be an artefact of how loss to follow‐up is measured: those who initiated earlier have had more chance to return to care [66]. As ART eligibility has expanded over time, this may partially explain the higher rates of attrition among healthier patients in this study, who initiated ART more recently.

Effect estimates were robust to a variety of sensitivity analyses. Youth were similar across clinics at baseline, so it is unsurprising that different methods to adjust for measured confounding resulted in similar results. Models using the secondary definition of the outcome showed greater effect sizes, suggesting more gaps in care among youth in general clinics.

Data for this analysis were from selected clinics, and may have overestimated attrition due to “silent transfers” [67, 68], when patients transfer to another clinic without informing the initiating clinic, and are recorded as new patients in the new facility. Data quality may differ between clinics, leading to differential misclassification of loss to follow‐up. Patients who initiated ART and became lost to follow‐up earlier had more chance to return to care, creating the potential for differential misclassification of the outcome. However, ART initiation era was similar between youth clinic and matched general clinic cohorts, and we also used secondary definition of loss to follow‐up, identifying the first gap in care, with similar results. We were not able to ascertain the timing of HIV infection. However, the median age of ART initiation was over 22 years for each clinic type, suggesting that perinatally acquired HIV was unlikely, and no different between groups. Self‐selection into youth clinics may have led to unmeasured confounding but strong confounder‐exposure and confounder‐attrition associations would be required to explain away the observed effect sizes. It is also possible that unmeasured confounding resulted in an underestimation of the true effect if youth‐targeted services attracted youth who face barriers to engagement in care and would not have initiated elsewhere.

## 5 CONCLUSIONS

Our analysis of observational data from Khayelitsha, South Africa, has attempted to disentangle the effects of several interventions for YLWH. Our results suggest that creating youth‐only spaces, training youth‐friendly counsellors and integrating primary healthcare services into youth adherence clubs can contribute to improving retention in this important group. Building on these results, future research could investigate these interventions as separate components of youth‐friendly services in other settings.

## COMPETING INTERESTS

The authors declare that they have no competing interests.

## AUTHORS’ CONTRIBUTIONS

VdA, PR, TD, CK, LTD and NZ were involved with developing and implementing the clinics. TC and MPF designed the research study. TC analysed the data and drafted the manuscript, with support from MPF, CRH and MC. CRH provided input on the study design, analysis and discussion. All authors have reviewed, provided input on and approved the final manuscript.

## FUNDING

MPF and CRH are supported by the Providence/Boston Center for AIDS Research (P30AI042853).

## Supporting information


**Figure S1**: Distribution of propensity scores by clinic type
**Figure S2**: Standardized differences before and after propensity score matching for each primary analysis
**Figure S3**: Post hoc power calculations for matched propensity score analyses for Youth Clinic A (N=2766, 833 events in primary analysis) and Youth Clinic B analyses (N=2598, 804 events in primary analysis)
**Table S1**: Missing values of WHO clinical stage, CD4 count and Regimen, by clinic type
**Table S2**: Baseline characteristics of ART patients who ever joined an adherence club before the age of 26, by clinic type
**Table S3**: Attrition by baseline characteristics and clinic type
**Table S4**: Youth Clinic A: summary of Cox regression results, E‐values and goodness of fit test results
**Table S5**: Youth Clinic B: summary of Cox regression results, E‐values and goodness of fit test results
**Table S6**: Numbers and proportions of patients with only one visit, loss to follow‐up after first visit, and meeting different definitions of the outcomes
**Table S7**: Results of covariate adjustment of primary models showing adjusted hazards ratios and % change
**Table S8**: Summary of Cox regression results when one day was added to each subjects' follow‐up time, and when no days were added to any subjects' follow‐up time, thereby excluding those who did not return after their first visit

## Data Availability

The data that support the findings of this study are available from the Provincial Health Data Centre (PHDC), Western Cape Government: Health. Data are available with the permission of the PHDC, Western Cape Government: Health: https://nhrd.health.gov.za.
