# Supplementary material for: Attrition from HIV care among youth initiating ART in youth‐only clinics compared with general primary healthcare clinics in Khayelitsha, South Africa: a matched propensity score analysis
Source: J Int AIDS Soc. 2022 Jan 25;25(1):e25854. doi: 10.1002/jia2.25854 (PMC8789247; doi:10.1002/jia2.25854)
Supplement: Supplementary file 1 — Figure S1: Distribution of propensity scores by clinic type Figure S2: Standardized differences before and after propensity score matching for each primary analysis Figure S3: Post hoc power calculations for matched propensity score analyses for Youth Clinic A (N=2766, 833 events in primary analysis) and Youth Clinic B analyses (N=2598, 804 events in primary analysis) Table S1: Missing values of WHO clinical stage, CD4 count and Regimen, by clinic type Table S2: Baseline characteristics of ART patients who ever joined an adherence club before the age of 26, by clinic type Table S3: Attrition by baseline characteristics and clinic type Table S4: Youth Clinic A: summary of Cox regression results, E‐values and goodness of fit test results Table S5: Youth Clinic B: summary of Cox regression results, E‐values and goodness of fit test results Table S6: Numbers and proportions of patients with only one visit, loss to follow‐up after first visit, and meeting different definitions of the outcomes Table S7: Results of covariate adjustment of primary models showing adjusted hazards ratios and % change Table S8: Summary of Cox regression results when one day was added to each subjects' follow‐up time, and when no days were added to any subjects' follow‐up time, thereby excluding those who did not return after their first visit [file JIA2-25-e25854-s001.docx]

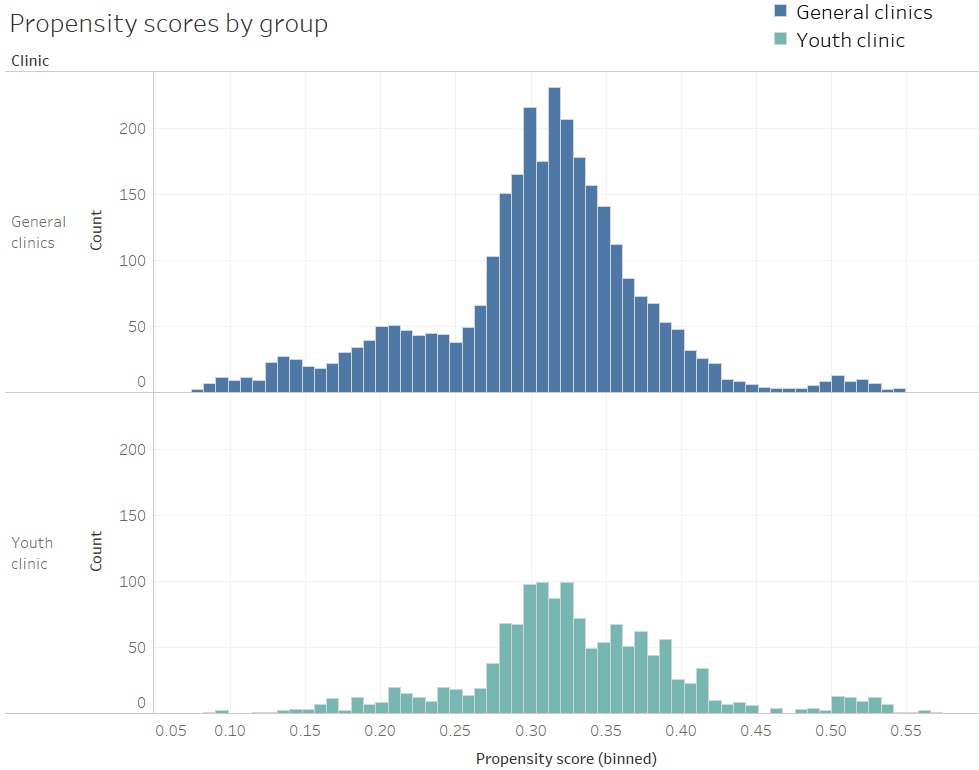

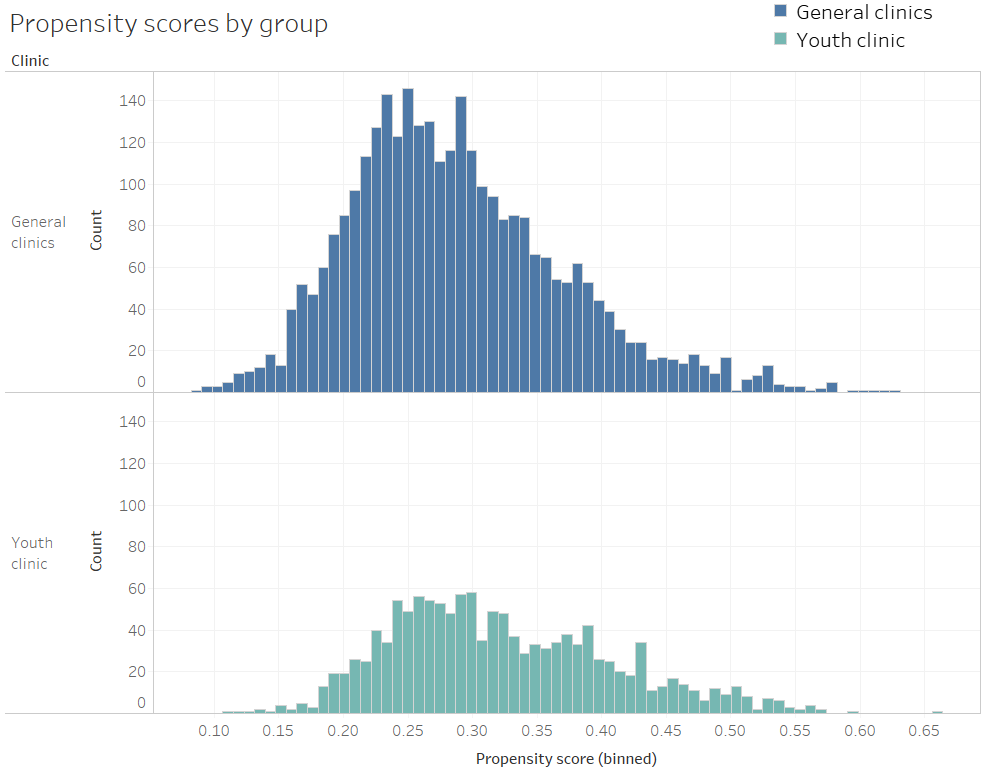


Supplementary figure 1 Distribution of propensity scores by clinic type

Supplementary figure 2 Standardized differences before and after propensity score matching for each primary analysis

Supplementary figure 3 Post hoc power calculations for matched propensity score analyses for Youth Clinic A (N=2766, 833 events in primary analysis) and Youth Clinic B analyses (N=2598, 804 events in primary analysis)

Supplementary table 1 Missing values of WHO clinical stage, CD4 count and Regimen, by clinic type

|  | N missing by clinic type | | | % missing by clinic type | | |
| --- | --- | --- | --- | --- | --- | --- |
|  | General clinics (N=3056) | Youth Clinic A (N=1383) | Youth Clinic B (N=1299) | General clinics (N=3056) | Youth Clinic A (N=1383) | Youth Clinic B (N=1299) |
| WHO stage | 247 | 74 | 108 | 8% | 5% | 8% |
| CD4 count | 367 | 116 | 117 | 12% | 8% | 9% |
| Regimen | 89 | 54 | 62 | 3% | 4% | 5% |

Supplementary table 2 Baseline characteristics of ART patients who ever joined an adherence club before the age of 26, by clinic type

|  | Youth Clinic A | Youth Clinic B | | General clinics | |
| --- | --- | --- | --- | --- | --- |
| N | 242 | 133 | | 537 | |
| Era of ART initiation |  |  | |  | |
| Eligibility CD4<200 (before August 2011) | 2% | 12% | | 3% | |
| Eligibility CD4<350 (Aug 2011-31 Dec 2014) | 19% | 56% | | 29% | |
| Eligibility <500(1 Jan 2015 - 31 Aug 2016) | 47% | 22% | | 52% | |
| All eligible (1 Sept 2016) | 32% | 11% | | 15% | |
| Age at first club visit |  | |  | |  |
| 12-17 years | 3% | 4% | | 3% | |
| 18-25 years | 97% | 96% | | 97% | |
| Median age (years) (IQR) | 22 (20.7-23.8) | 22.9 (21.4-24.5) | | 23.8 (22-24.9) | |
| Sex |  |  | |  | |
| Male | 10% | 7% | | 11% | |
| WHO stage at ART initiation |  | |  | |  |
| Stage 1 | 80% | 74% | | 75% | |
| Stage 2 | 11% | 13% | | 14% | |
| Stage 3 | 3% | 9% | | 4% | |
| Stage 4 | 0% | 1% | | 1% | |
| Stage 2-4 | 14% | 23% | | 19% | |
| Stage missing | 6% | 4% | | 6% | |
| CD4 Count at ART initiation (cells/mm3) |  | |  | |  |
| <200 | 11% | 13% | | 14% | |
| 200-349 | 31% | 36% | | 34% | |
| 350-500 | 31% | 29% | | 29% | |
| >500 | 21% | 12% | | 17% | |
| Missing | 6% | 10% | | 7% | |
| Median CD4 count (IQR) | 368 (287-477) | 335.5 (229.5-456.5) | | 344.5 (250-459) | |
| EFV-free regimens | 1% | 8% | | 2% | |
| TDF-free regimens | 1% | 4% | | 1% | |
| Missing regimen information | 5% | 3% | | 1% | |
| Median months in club (before age 26) | 11.7 (4.6-21.1) | 10.1 (1.8-20.5) | | 16.7 (6.3-25.8) | |
| Median months on ART before club | 9.7 (3.3-20) | 24.8 (15.5-43.6) | | 15.3 (8.6-25.3) | |

Supplementary table 3 Attrition by baseline characteristics and clinic type

|  | **6-month attrition†** | | | | **12-month attrition‡** | | |
| --- | --- | --- | --- | --- | --- | --- | --- |
|  | Youth Clinic A | Youth Clinic B | | General Clinics | Youth Clinic A | Youth Clinic B | General Clinics |
|  | N=1225 | | N=1129 | N=2803 | N=1039 | N=978 | N=2523 |
| Total | 15% | | 17% | 21% | 22% | 24% | 27% |
| Sex |  | |  |  |  |  |  |
| Male | 15% | | 16% | 19% | 18% | 23% | 26% |
| Female | 15% | | 17% | 21% | 22% | 24% | 28% |
| Era of ART initiation (by CD4 count eligibility criteria) | | | | |  |  |  |
| <200 (before August 2011) | 7% | | 11% | 13% | 18% | 18% | 17% |
| <350 (Aug 2011-31 Dec 2014) | 13% | | 14% | 18% | 20% | 20% | 24% |
| <500 (1 Jan 2015 - 31 Aug 2016) | 16% | | 16% | 21% | 23% | 26% | 29% |
| All eligible (After 1 Sept 2016) | 23% | | 28% | 30% | 28% | 35% | 38% |
| Age |  | |  |  |  |  |  |
| 12-17 years | 13% | | 16% | 18% | 19% | 21% | 24% |
| 18-25 years | 15% | | 17% | 22% | 22% | 24% | 28% |
| WHO stage at initiation | | | |  |  |  |  |
| Stage 1 | 16% | | 18% | 21% | 23% | 26% | 28% |
| Stage 2 | 8% | | 10% | 17% | 11% | 12% | 23% |
| Stage 3 | 15% | | 10% | 15% | 20% | 16% | 20% |
| Stage 4 | 25% | | 23% | 24% | 43% | 26% | 28% |
| Stage 2-4 | 10% | | 12% | 17% | 14% | 16% | 22% |
| Stage missing | 26% | | 23% | 32% | 34% | 32% | 38% |
| Baseline CD4 Count | | |  |  |  |  |  |
| <200 | 13% | | 13% | 18% | 18% | 21% | 23% |
| 200-350 | 13% | | 13% | 20% | 21% | 17% | 26% |
| 350-500 | 17% | | 20% | 23% | 22% | 29% | 31% |
| >500 | 21% | | 23% | 24% | 29% | 26% | 32% |
| CD4 count missing | 17% | | 21% | 20% | 23% | 40% | 27% |
| ART Regimen at initiation | | | |  |  |  |  |
| EFV-free regimens | 4% | | 11% | 16% | 10% | 15% | 18% |
| TDF-free regimens | 10% | | 11% | 13% | 17% | 17% | 18% |
| TDF-EFV regimens | 16% | | 18% | 22% | 22% | 25% | 28% |
| missing regimen information | 28% | | 17% | 35% | 36% | 21% | 47% |
| Ever in adherence club at clinic | 1% | | 1% | 0% | 3% | 2% | 2% |
| Never in adherence club at clinic | 19% | | 19% | 27% | 27% | 28% | 35% |
| ^†^ 6-month retention is only presented for those who initiate ART more than nine months before dataset closure  ^‡^12-month retention is only presented for those who initiate ART more than 15 months before dataset closure | | | | | | | |

Supplementary table 4 Youth Clinic A: summary of Cox regression results, e-values and goodness of fit test results

| Method | Variables adjusted for/included in propensity score model | HR of risk of attrition in youth clinic vs. general clinics (95% CI) | Person time in years  (events) | Goodness of fit using Schoenfeld residuals (p-value) | e-values  (e-value of upper CI limit) |
| --- | --- | --- | --- | --- | --- |
| Full cohort Analyses | | | | |  |
| Matched propensity score approach^†^ | start date and WHO disease stage | 0.81 (0.70–0.93) | 4367 (833) | 0.03 | 0.63 (0.77) |
|  | guideline era and WHO disease stage | 0.74 (0.65–0.85) | 4290 (862) | 0.02 | 0.57 (0.67) |
| No matching, combined Cox model including Youth Clinic A & B^‡^ | crude | 0.78 (0.69–0.88) | 8896 (1759) | 0.04 | 0.60 (0.71) |
|  | adjusting for start date | 0.80 (0.71–0.91) | 8896 (1759) | 0.16 | 0.63 (0.74) |
|  | adjusting for ART start date, WHO stage | 0.81 (0.72–0.92) | 8896 (1759) | 0.43 | 0.64 (0.76) |
| Secondary definition of outcome^§^ | | |  |  |  |
| Matched propensity score approach^†^ | start date and WHO disease stage | 0.68 (0.60–0.76) | 3770 (1091) | 0.55 | 0.51 (0.59) |
|  | guideline era and WHO disease stage | 0.62 (0.55–0.70) | 3689 (1122) | 0.35 | 0.47 (0.53) |
| No matching, combined Cox model including Youth Clinic A & B^†^ | crude | 0.66 (0.59–0.73) | 7708 (2312) | 0.96 | 0.50 (0.56) |
|  | adjusting for start date | 0.67 (0.60–0.75) | 7708 (2312) | 0.88 | 0.51 (0.57) |
|  | adjusting for ART start date, WHO stage | 0.68 (0.61–0.75) | 7708 (2312) | 0.6 | 0.51 (0.58) |
| Adherence club patients only | | | | |  |
| No matching, combined Cox model including Youth Clinic A & B^‡^ | crude | 0.56 (0.32–0.96) | 970.57 (95) | 0.55 | 0.42 (0.84) |
|  | adjusting for start date | 0.49 (0.28–0.84) | 970.57 (95) | 0.15 | 0.37 (0.67) |
|  | adjusting for ART start date, WHO stage | 0.49 (0.28–0.85) | 970.57 (95) | 0.31 | 0.38 (0.67) |
|  | adjusting for ART start date, WHO stage and age at club start | 0.50 (0.29–0.88) | 970.57 (95) | 0.26 | 0.38 (0.71) |
|  | adjusting for ART start date, WHO stage, age at club start and time on ART at club start | 0.48 (0.28–0.85) | 970.57 (95) | 0.19 | 0.37 (0.67) |
| Secondary definition of outcome^§^ | | | | |  |
| No matching, combined Cox model including Youth Clinic A & B^‡^ | crude | 0.50 (0.30–0.85) | 895.46 (99) | 0.65 | 0.38 (0.68) |
|  | adjusting for start date | 0.48 (0.28–0.83) | 895.46 (99) | 0.02 | 0.37 (0.65) |
|  | adjusting for ART start date, WHO stage | 0.49 (0.29–0.85) | 895.46 (99) | 0.73 | 0.38 (0.67) |
|  | adjusting for ART start date, WHO stage and age at club start | 0.49 (0.28–0.84) | 895.46 (99) | 0.7 | 0.38 (0.66) |
|  | adjusting for ART start date, WHO stage, age at club start and time on ART at club start | 0.48 (0.28–0.82) | 895.46 (99) | 0.73 | 0.37 (0.64) |
| ^†^Analyses conducted separately for Youth Clinic A and B.  ^‡^Analysis of full dataset including Youth Clinic A and B, with general clinics as reference group  ^§^the first time there is a nine-month gap in care patients are considered lost to follow-up even if they return to care, with the date of last visit before the gap in care being the outcome date | | | | | |

Supplementary table 5 Youth Clinic B: summary of Cox regression results, e-values and goodness of fit test results

| Method | Variables adjusted for/included in propensity score model | HR of risk of attrition in youth clinic vs. general clinics (95% CI) | Person time in years  (events) | Goodness of fit using Schoenfeld residuals (p-value) | e-values  (e-value of upper CI limit) |
| --- | --- | --- | --- | --- | --- |
| Full cohort Analyses | | | | |  |
| Matched propensity score approach^†^ | start date and WHO disease stage | 0.85 (0.74–0.98) | 4341 (804) | 0.16 | 0.68 (0.88) |
|  | guideline era and WHO disease stage | 0.90 (0.78–1.03) | 4388 (787) | 0.17 | 0.73 (1) |
| No matching, combined Cox model including Youth Clinic A & B^‡^ | crude | 0.80 (0.71–0.90) | 8896 (1759) | 0.04 | 0.62 (0.73) |
|  | adjusting for start date | 0.86 (0.76–0.97) | 8896 (1759) | 0.16 | 0.69 (0.86) |
|  | adjusting for ART start date, WHO stage | 0.87 (0.77–0.98) | 8896 (1759) | 0.43 | 0.70 (0.89) |
| Secondary definition of outcome^§^ | | |  |  |  |
| Matched propensity score approach^†^ | start date and WHO disease stage | 0.72 (0.64–0.81) | 3796 (1064) | 0.7 | 0.55 (0.63) |
|  | guideline era and WHO disease stage | 0.75 (0.66–0.85) | 3824 (1044) | 0.89 | 0.58 (0.67) |
| No matching, combined Cox model including Youth Clinic A & B^†^ | crude | 0.68 (0.61–0.75) | 7708 (2312) | 0.96 | 0.51 (0.58) |
|  | adjusting for start date | 0.71 (0.64–0.79) | 7708 (2312) | 0.88 | 0.54 (0.61) |
|  | adjusting for ART start date, WHO stage | 0.72 (0.64–0.80) | 7708 (2312) | 0.6 | 0.55 (0.62) |
| Adherence club patients only | | | | |  |
| No matching, combined Cox model including Youth Clinic A & B^‡^ | crude | 0.83 (0.48–1.45) | 970.57 (95) | 0.55 | 0.66 (1.00) |
|  | adjusting for start date | 0.98 (0.56–1.72) | 970.57 (95) | 0.15 | 0.88 (1.00) |
|  | adjusting for ART start date, WHO stage | 0.98 (0.56–1.72) | 970.57 (95) | 0.31 | 0.88 (1.00) |
|  | adjusting for ART start date, WHO stage and age at club start | 1.00 (0.57–1.77) | 970.57 (95) | 0.26 | 0.96 (1.00) |
|  | adjusting for ART start date, WHO stage, age at club start and time on ART at club start | 1.07 (0.60–1.90) | 970.57 (95) | 0.19 | 0.78 (1.00) |
| Secondary definition of outcome^§^ | | | | |  |
| No matching, combined Cox model including Youth Clinic A & B^‡^ | crude | 0.58 (0.32–1.06) | 895.46 (99) | 0.65 | 0.44 (1.00) |
|  | adjusting for start date | 0.61 (0.33–1.12) | 895.46 (99) | 0.02 | 0.46 (1.00) |
|  | adjusting for ART start date, WHO stage | 0.60 (0.33–1.10) | 895.46 (99) | 0.73 | 0.46 (1.00) |
|  | adjusting for ART start date, WHO stage and age at club start | 0.60 (0.32–1.09) | 895.46 (99) | 0.7 | 0.45 (1.00) |
|  | adjusting for ART start date, WHO stage, age at club start and time on ART at club start | 0.63 (0.34–1.17) | 895.46 (99) | 0.73 | 0.48 (1.00) |
| ^†^Analyses conducted separately for Youth Clinic A and B.  ^‡^Analysis of full dataset including Youth Clinic A and B, with general clinics as reference group  ^§^the first time there is a nine-month gap in care patients are considered lost to follow-up even if they return to care, with the date of last visit before the gap in care being the outcome date | | | | | |

Supplementary table 6 Numbers and proportions of patients with only one visit, LTFU after first visit, and meeting different definitions of the outcomes

|  | Youth Clinic A | General clinics matches | Youth Clinic B | General clinic matches |
| --- | --- | --- | --- | --- |
| N | 1383 | 1383 | 1299 | 1299 |
| No follow-up time, N | 86 | 92 | 97 | 86 |
| No follow-up time, % | 6% | 7% | 7% | 7% |
| Immediately LTFU, N | 62 | 68 | 33 | 70 |
| Immediately LTFU, % | 4% | 5% | 3% | 5% |
| primary definition of outcome, N | 365 | 468 | 357 | 447 |
| primary definition of outcome, % | 26% | 34% | 27% | 34% |
| Secondary definition of outcome (any 9-month gap in care), N | 445 | 646 | 437 | 627 |
| Secondary definition of outcome (any 9-month gap in care), % | 32% | 47% | 34% | 48% |
| Had gap in care but returned before end of follow-up period, N | 80 | 178 | 80 | 180 |
| Had gap in care but returned before end of follow-up period, % | 6% | 13% | 6% | 14% |

Supplementary table 7 Results of covariate adjustment of primary models showing adjusted hazards ratios and % change

|  | Youth Clinic A (HR: 0.807) | | Youth Clinic B (HR: 0.853) | |
| --- | --- | --- | --- | --- |
| Covariate | adjusted HR  (clinic on attrition) | % change | adjusted HR  (clinic on attrition) | % change |
| WHO stage (continuous) | 0.807 | 0.03% | 0.856 | -0.38% |
| WHO stage (indicator vaiable) | 0.818 | -1.28% | 0.853 | 0.01% |
| WHO stage (stage 1 vs >1) | 0.810 | -0.37% | 0.855 | -0.33% |
| Baseline CD4 count | 0.811 | -0.45% | 0.848 | 0.56% |
| Baseline CD4 count (categorised) | 0.819 | -1.41% | 0.849 | 0.46% |
| Age (continuous) | 0.810 | -0.35% | 0.851 | 0.16% |
| Age (<18 yrs vs >18 yrs) | 0.807 | 0.07% | 0.853 | -0.01% |
| Year of ART initiation (indicator variable) | 0.808 | -0.16% | 0.845 | 0.91% |
| Year of ART initiation (continuous) | 0.805 | 0.28% | 0.850 | 0.34% |
| Started ART Aug 2011-31 Dec 2014 | 0.803 | 0.51% | 0.844 | 1.01% |
| Started ART 1 Jan 2015 - 31 Aug 2016 | 0.806 | 0.16% | 0.853 | 0.01% |
| Started ART after 31 Aug 2016 | 0.807 | 0.07% | 0.847 | 0.64% |
| ART start date | 0.805 | 0.32% | 0.850 | 0.32% |
| Sex (male vs female) | 0.807 | 0.03% | 0.853 | -0.02% |
| Propensity score | 0.808 | -0.11% | 0.844 | 1.00% |

Supplementary table 8 Summary of Cox regression results when one day was added to each subjects’ follow-up time, and when no days were added to any subjects’ follow-up time, thereby excluding those who did not return after their first visit

| Method | Variables adjusted for/included in propensity score model | HR of risk of attrition in youth clinic vs. general clinics (95% CI) | | | | | | |
| --- | --- | --- | --- | --- | --- | --- | --- | --- |
|  |  | Original | | | | One day added to all | No days added | |
| **Youth Clinic A analysis** | | | | | | | | |
| Matched propensity score approach | start date and WHO disease stage | | | 0.81 (0.70–0.93) | | 0.81 (0.71-0.92) | 0.79 (0.68-0.91) | |
|  | guideline era and WHO disease stage | | | 0.74 (0.65–0.85) | | 0.74 (0.65-0.84) | 0.74 (0.64-0.86) | |
| No matching, combined Cox model including Youth Clinic A & B | crude | | | 0.78 (0.69–0.88) | | 0.78 (0.69-0.88) | 0.78 (0.68-0.88) | |
|  | adjusting for start date | | | 0.80 (0.71–0.91) | | 0.80 (0.71-0.91) | 0.80 (0.70-0.91) | |
|  | adjusting for ART start date, WHO stage | | | 0.81 (0.72–0.92) | | 0.81 (0.72-0.92) | 0.80 (0.70-0.92) | |
| Secondary definition of outcome | | | | | |  |  | |
| Matched propensity score approach | start date and WHO disease stage | | | 0.68 (0.60–0.76) | | 0.68 (0.60-0.76) | 0.68 (0.59-0.77) | |
|  | guideline era and WHO disease stage | | | 0.62 (0.55–0.70) | | 0.62 (0.55-0.70) | 0.64 (0.56-0.72) | |
| No matching, combined Cox model including Youth Clinic A & B | crude | | | 0.66 (0.59–0.73) | | 0.66 (0.59-0.73) | 0.66 (0.59-0.75) | |
|  | adjusting for start date | | | 0.67 (0.60–0.75) | | 0.67 (0.60-0.75) | 0.68 (0.60-0.76) | |
|  | adjusting for ART start date, WHO stage | | | 0.68 (0.61–0.75) | | 0.68 (0.61-0.75) | 0.69 (0.61-0.77) | |
| **Youth Clinic B analysis** | | | | | | | | |
| Matched propensity score approach | start date and WHO disease stage | | 0.85 (0.74–0.98) | | | 0.85 (0.74-0.98) | | 0.92 (0.79-1.06) |
|  | guideline era and WHO disease stage | | 0.90 (0.78–1.03) | | | 0.90 (0.78-1.03) | | 0.96 (0.82-1.11) |
| No matching, combined Cox model including Youth Clinic A & B | crude | | 0.80 (0.71–0.90) | | | 0.80 (0.71-0.90) | | 0.85 (0.75-0.97) |
|  | adjusting for start date | | 0.86 (0.76–0.97) | | | 0.86 (0.76-0.97) | | 0.92 (0.81-1.04) |
|  | adjusting for ART start date, WHO stage | | 0.87 (0.77–0.98) | | | 0.87 (0.77-0.98) | | 0.92 (0.81-1.05) |
| Secondary definition of outcome | | | | | |  | |  |
| Matched propensity score approach | start date and WHO disease stage | | | | 0.72 (0.64–0.81) | 0.72 (0.64-0.81) | | 0.78 (0.68-0.89) |
|  | guideline era and WHO disease stage | | | | 0.75 (0.66–0.85) | 0.75 (0.66-0.85) | | 0.81 (0.71-0.93) |
| No matching, combined Cox model including Youth Clinic A & B | crude | | | | 0.68 (0.61–0.75) | 0.68 (0.61-0.75) | | 0.72 (0.65-0.81) |
|  | adjusting for start date | | | | 0.71 (0.64–0.79) | 0.71 (0.64-0.79) | | 0.76 (0.68-0.86) |
|  | adjusting for ART start date, WHO stage | | | | 0.72 (0.64–0.80) | 0.72 (0.64-0.80) | | 0.77 (0.68-0.86) |
